# Intracranial angiomatoid fibrous histiocytoma in a 2-month-old infant: a rare case report

**DOI:** 10.3389/fonc.2026.1679314

**Published:** 2026-06-15

**Authors:** Xiaoyu Yang, Xiaofeng Chen, Yi Zhang, Yanming Ren, Junwen Guan, Ke Wu

**Affiliations:** 1 Department of Neurosurgery, West China Hospital, Sichuan University, Chengdu, Sichuan, China; 2 Department of Neurosurgery, Chengdu Women’s and Children’s Central Hospital, School of Medicine, University of Electronic Science and Technology of China, Chengdu, China; 3 Health Management Center, West China Hospital, Sichuan University, Chengdu, Sichuan, China; 4 Department of Neurosurgery, Xichang Peoples’ Hospital, Liangshan, Sichuan, China

**Keywords:** angiomatoid fibrous histiocytoma, infant, intracranial, intracranial tumor, skull and brain

## Abstract

**Background:**

Angiomatoid fibrous histiocytoma (AFH) is a rare tumor typically arising in the extremities or trunk. Its intracranial occurrence in young infants constitutes a distinct rarity that poses significant clinical challenges. This report details the diagnosis and management of AFH in a 2-month-old infant with an intracranial lesion.

**Case description:**

A 2-month-old female infant presented with a postauricular mass. Magnetic resonance imaging (MRI) and computed tomography (CT) confirmed an intracranial tumor. Subsequent craniotomy revealed a 4 × 3 × 4 cm epidural mass between the dura mater and galea aponeurotica. Histopathology confirmed a rare intracranial AFH.

**Conclusion:**

Intracranial AFH in infants is exceedingly rare, requiring definitive diagnosis through immunohistochemistry and molecular genetic testing. Complete surgical resection represents the gold-standard management; however, limited experience in treating very young infants necessitates meticulous intraoperative hemostasis and robust collaboration with pathologists.

## 1 Introduction

Angiomatoid fibrous histiocytoma (AFH) is a rare mesenchymal tumor predominantly affecting children and young adults (1). While generally low-grade malignant, some cases exhibit aggressive behavior. Histopathologically, it manifests as ill-defined nodular lesions encapsulated by a fibrous pseudocapsule with peripheral lymphoid cuffing. The tumor core demonstrates polymorphic proliferation of histiocytes, multinucleated giant cells, and mixed inflammatory infiltrates. These neoplasms primarily involve the extremities and trunk, with rare extra-compartmental occurrence (2). We present a 2-month-old female infant hospitalized for a left postauricular mass discovered 1 week prior. Computed tomography (CT) and magnetic resonance imaging (MRI) confirmed an intracranial tumor, successfully excised via craniotomy. Definitive histopathological diagnosis established AFH. This case aims to enhance recognition of intracranial AFH and inform the management of infantile intracranial tumors.

## 2 Case report

### 2.1 General information

This pediatric patient was admitted for a 1-week history of a postauricular mass. One week prior to admission, the parents palpated a firm, non-tender left postauricular mass without surrounding erythema or edema. Palpation elicited no irritability. Initial evaluation at a local hospital included contrast-enhanced CT, suggesting an intracranial neoplasm, prompting referral to our tertiary center. The parents subsequently presented to our outpatient clinic and proceeded with inpatient admission. Obstetric history revealed an uncomplicated pregnancy without maternal exposure to teratogens during the perinatal period. The infant was delivered via spontaneous vaginal delivery at 38 5/7 weeks’ gestation.

### 2.2 Physical examination

The infant appeared well-nourished with age-appropriate developmental milestones. She was alert and conscious with normal skin coloration, exhibiting pink mucous membranes and nail beds without petechiae or ecchymoses. Oropharyngeal examination revealed non-erythematous mucosa and non-enlarged tonsils. The anterior fontanelle was patent with normal tension. A 2-cm firm, non-mobile, well-defined mass was palpated posterior to the left auricle, surrounded by non-inflamed skin. Auscultation demonstrated clear breath sounds bilaterally. Cardiac assessment showed regular rhythm without murmurs. Abdominal examination disclosed a soft, non-tender abdomen without rebound tenderness; hepatic and splenic margins remained non-palpable.

### 2.3 Laboratory examination

Complete blood count revealed no significant abnormalities: hemoglobin, 140 g/L; white blood cells, 5 × 10⁹/L. Biochemical profile demonstrated normal hepatic and renal function with unremarkable electrolyte levels. Coagulation studies showed no abnormalities. Serum tumor markers were within normal limits. Stool and urinalysis findings were noncontributory.

### 2.4 Imaging examination

CT: Non-contrast CT reveals a well-demarcated, isodense mass (4.2 × 2.9 × 3.6 cm) in the left temporo-occipital region. Adjacent calvarial defect is noted. Mass effect is evident with compression of the left temporo-occipital lobe and cerebellar hemisphere. No abnormal parenchymal densities are observed (**Figures 1A, B****).** MRI: T1-weighted MRI demonstrates an irregular heterogenous mass with predominant hypointensity. T2 sequences show predominant hyperintensity. Post-contrast imaging reveals heterogeneous enhancement and adjacent dural thickening. The lesion is supplied by branches of the occipital and superficial temporal arteries, exerting mass effect on the superior petrosal, transverse, and sigmoid sinuses (**Figures 1C, D****).**

**Figure 1:**
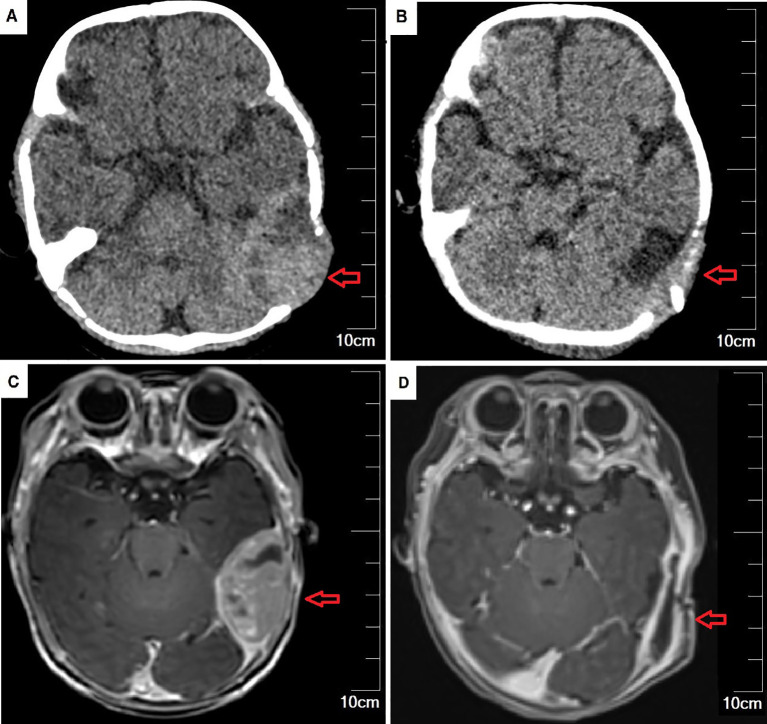
Neuroimaging of intracranial tumors. **(A)** Preoperative intracranial CT revealed a well-demarcated tumor in the left temporo-occipital region with bony destruction. The red arrow indicates the tumor. **(B)** Postoperative day 1 head CT confirmed complete resection of the left temporo-occipital tumor with a surgical site bony defect and no evidence of hemorrhage. The red arrow denotes the operative bed. **(C)** Preoperative head MRI revealed a well-circumscribed, heterogeneously enhancing tumor in the left temporo-occipital region with heterogeneous signal intensity. The red arrow denotes the lesion. **(D)** One-month postoperative brain MRI demonstrated complete tumor resection in the left temporo-occipital region with partial restoration of previously compressed venous sinus flow. The red arrow denotes the operative bed.

### 2.5 Surgical procedure

Following preoperative diagnosis of an intracranial tumor based on clinical history, physical examination, and ancillary investigations, a craniotomy was performed under general anesthesia with the patient in right lateral decubitus position. After standard preparation and draping, a linear incision was made over the left temporo-occipital mass. Dissection through skin and muscle layers with electrocautery and bipolar coagulation achieved hemostasis, exposing the tumor and calvarium. Craniectomy using rongeurs removed overlying bone, fully revealing a 4 × 3 × 4 cm firm, variegated red–white epidural tumor between the dura mater and galea aponeurotica, with invasion into calvarium, dura, sigmoid sinus, transverse sinus, and superior petrosal sinus. Based on the intraoperative findings, we believe that the lesion most likely originates from the dura mater. Microscopic gross-total resection was accomplished, followed by venous sinus repair (sinorrhaphy), watertight dural closure, and hemostasis. Closure included layered reapproximation of muscle, galea, and skin after placement of an extradural drain.

### 2.6 Pathological findings

Gross examination revealed a 4 × 3 × 4 cm variegated red–gray mass with similar cut surface characteristics (**Figure 2****).** Histopathologic analysis demonstrated a spindle cell neoplasm arranged in diffuse sheets and short fascicles, interspersed with scattered multinucleated giant cells exhibiting mild-to-moderate nuclear atypia without definitive mitotic activity. Immunohistochemistry showed the following: ALK(−), MyoD1(−), Myogenin(−), MUC4(−), CD163(+), CD1a(−), WT-1(−), CD99(+), STAT6(−), S100(−), ATRX(−), Langerin(−), BRAF V600E(−), EMA(+), Desmin(+), and CD68(+) (**Figure 3**). Molecular testing confirmed EWSR1::CREB1 gene fusion. Final pathological diagnosis: Intracranial angiomatoid fibrous histiocytoma (CNS WHO grade 1).

**Figure 2:**
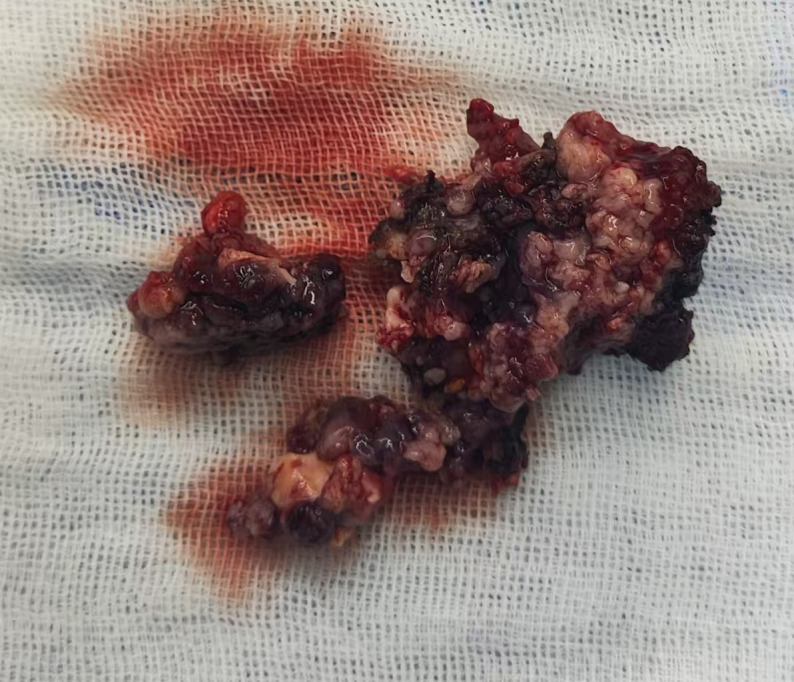
Intraoperatively resected intracranial tumor tissue.

**Figure 3:**
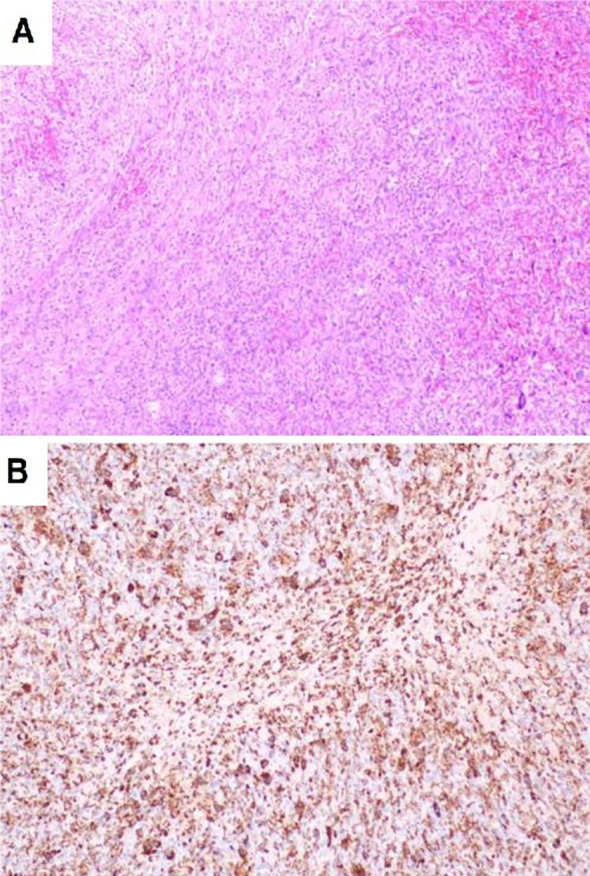
Histopathological micrograph of tumor tissue. **(A)** H&E-stained sections demonstrate a predominant proliferation of spindled tumor cells arranged in diffuse sheets and short fascicular patterns. **(B)** Scattered multinucleated giant cells are highlighted on immunohistochemical staining for CD68.

### 2.7 Postoperative follow-up

At the 1-month postoperative follow-up, the infant demonstrated satisfactory wound healing without neurological deficits and exhibited age-appropriate developmental progression. Surveillance brain MRI revealed minimal subdural fluid collection at the resection site, no evidence of residual tumor, and partial restoration of flow in previously compressed dural venous sinuses. Continued surveillance with MRI every 6 months was recommended, with cranioplasty to be considered after 2 years pending assessment of calvarial regeneration.

## 3 Discussion

AFH is a rare, potentially low-grade malignant tumor predominantly occurring in the extremities or trunk soft tissues of children/adolescents. Its histopathologic hallmark is a triphasic pattern: blood-filled pseudovascular spaces (lacking endothelial lining), solid sheets of histiocyte-like/spindle cells, and a thick fibrous pseudocapsule with prominent lymphoid follicles (3). Molecularly, >90% demonstrate characteristic EWSR1::CREB1 or EWSR1::ATF1 gene fusions (4). Complete surgical excision is the primary treatment, typically without adjuvant therapy. Despite its malignant classification, AFH exhibits indolent biological behavior; complete resection confers favorable prognosis with low local recurrence (<5%) and rare distant metastasis (5).

Intracranial AFH is a rare meningeal tumor. Its typical imaging features include a well-defined, round-like supratentorial mass of meningeal origin (6). On non-contrast CT, it appears isodense or slightly hyperdense. On MRI, T2-weighted images often show heterogeneous high signal intensity, with post-contrast scans demonstrating obvious homogeneous enhancement and a possible “dural tail sign”. Peritumoral edema is usually mild (7). The main differential diagnoses include meningioma, solitary fibrous tumor/hemangiopericytoma, and metastatic tumors. Definitive diagnosis ultimately relies on pathological examination and molecular testing.

“Intracranial mesenchymal tumor with FET-CREB fusion” is a newly established classification of central nervous system tumors defined by its molecular characteristics. The core diagnostic criterion is the detection of a fusion between the FET gene (primarily EWSR1) and a CREB family gene (such as CREB1 or ATF1) (8). This classification resolves previous diagnostic challenges for intracranial spindle cell tumors, clearly distinguishing them from morphologically similar entities like meningiomas and solitary fibrous tumors. These tumors predominantly occur in the meninges and are frequently observed in children and adolescents (6). Radiologically, they often present as well-defined, enhancing masses (7). Histologically, they typically exhibit spindle cells arranged in fascicles accompanied by a rich vascular network. Their clinical behavior is mostly indolent, and prognosis is generally favorable after gross total resection, though recurrence is possible, making long-term follow-up essential (9). The establishment of this molecularly defined entity marks a significant shift in diagnosis from reliance on morphology to the integration of molecular features, holding important clinical value for achieving precise diagnosis, guiding treatment decisions, and assessing prognosis.

This study has the following limitations: the follow-up period is relatively short, making it difficult to assess the long-term recurrence risk and prognosis of the tumor. Additionally, constrained by practical conditions, more comprehensive molecular analyses (such as large-panel sequencing and methylation testing) were not conducted, which may affect the precise molecular subtyping of the case. In the future, extending follow-up and improving testing are needed to accumulate more data and deepen the understanding of this disease.

This case exhibits unique features: First, AFH predominantly arises in extremities/trunk, making intracranial occurrence exceptionally rare—particularly in a 2-month-old infant, representing the youngest reported case per literature review. Second, the tumor’s intracranial epicenter with calvarial penetration and dural sinus invasion mandated craniectomy (vs. craniotomy) for exposure, a hemorrhage-avoidance strategy critical in neonates with limited blood volume. Third, deliberate deferral of cranioplasty addressed infant-specific challenges: thin calvarium precluding inlay grafts, screw fixation risks for onlay prostheses, and potential calvarial regeneration obviating secondary repair.

## 4 Conclusions

Infantile intracranial AFH constitutes an exceedingly rare pathological entity whose definitive diagnosis necessitates immunohistochemical profiling and molecular genetic testing. Complete surgical resection represents the optimal therapeutic strategy for this condition.

The neurosurgical management of intracranial tumors in very young infants presents unique challenges due to limited clinical experience, necessitating meticulous surgical planning with paramount emphasis on hemostasis control and robust multidisciplinary pathological collaboration.

## 5 Patient’s perspective

The parents expressed profound distress regarding their child’s intracranial tumor, noting complete unfamiliarity with this rare neoplasm, while conveying deepest gratitude for the successful surgical intervention performed on their 2-month-old infant. Written informed consent for publication was obtained from the patient’s legal guardians.

## Data Availability

The original contributions presented in the study are included in the article/supplementary material. Further inquiries can be directed to the corresponding author.
